# Multiclass portfolio optimization via variational quantum Eigensolver with Dicke state ansatz

**DOI:** 10.1038/s41598-026-36333-4

**Published:** 2026-02-13

**Authors:** J. V. S. Scursulim, Gabriel M. Langeloh, Victor L. Beltran, Samuraí Brito

**Affiliations:** Instituto de Ciência e Tecnologia Itaú, São Paulo, Brazil

**Keywords:** Mathematics and computing, Physics

## Abstract

Combinatorial optimization is a fundamental challenge in various domains, with portfolio optimization standing out as a key application in finance. Despite numerous quantum algorithmic approaches proposed for this problem, most overlook a critical feature of realistic portfolios: diversification. In this work, we introduce a novel quantum framework for multiclass portfolio optimization that explicitly incorporates diversification by leveraging multiple parametrized Dicke states, simultaneously initialized to encode the diversification constraints, as an ansatz of the Variational Quantum Eigensolver. A key strength of this ansatz is that it initializes the quantum system in a superposition of only feasible states, inherently satisfying the constraints. This significantly reduces the search space and eliminates the need for penalty terms. In addition, we also analyze the impact of different classical optimizers in this hybrid quantum-classical approach. Our findings demonstrate that, when combined with the CMA-ES optimizer, the Dicke state ansatz achieves superior performance in terms of convergence rate, approximation ratio, and measurement probability. These results underscore the potential of this method to solve practical, diversification-aware portfolio optimization problems relevant to the financial sector.

## Introduction

Quantum Computing (QC) represents a groundbreaking technology poised to solve problems that lie beyond the capabilities of classical computers. It operates by manipulating quantum systems and harnessing unique properties such as superposition, interference, and entanglement. This approach enables the execution of advanced algorithms designed to address complex challenges with remarkable efficiency. The potential of quantum computing spans multiple fields, promising significant impacts across industries and scientific disciplines; some of them include: quantum chemistry and material science^1–3^, machine learning^4–9^, finance^10–17^, and optimization^18–24^.

Optimization plays a fundamental role across various domains, with classic examples including the traveling salesman problem^25^, vehicle routing^26^, bin packing^27^, and portfolio optimization^28^-the central focus of this article. These problems are traditionally tackled using classical techniques such as mixed-integer programming^29^, approximation algorithms and metaheuristics^30^, and neural networks^31,32^. However, due to their computational complexity, these methods tend to have limitations in either their running time or solution quality as the number of variables increases.

Portfolio optimization exemplifies this challenge, especially in multiclass scenarios that require allocation across diverse asset categories (e.g., equities, bonds, commodities). While classical approaches like the Markowitz mean-variance model^33^ perform well for small-scale instances, multiclass formulations introduce combinatorial and equality constraints that lead to mixed-integer quadratic programs (MIQPs), which are hard to solve optimally, particularly in real-time or resource-limited settings.

In response to these limitations, Variational Quantum Algorithms (VQAs)^34,35^ have emerged as promising tools for near-term quantum advantage. Designed for Noisy Intermediate-Scale Quantum (NISQ) devices, VQAs, especially the Variational Quantum Eigensolver (VQE)^36^, have demonstrated potential in solving combinatorial problems by mapping them to Ising Hamiltonians and approximating the ground state via parameterized quantum circuits. The effectiveness of this approach heavily depends on the choice of ansatz, particularly when handling complex constraints such as those in multiclass portfolio optimization.

To that end, this paper proposes the use of a Dicke state-based ansatz within the VQE framework to efficiently handle multiclass portfolio optimization, incorporating a realistic feature in this problem related to diversification of the investment. Our contributions are threefold: (i) *Modeling advantage* - we formulate a multiclass portfolio optimization problem suitable for quantum encoding by introducing a parameterized Dicke-state ansatz, in which diversification constraints are inherently satisfied through state preparation, eliminating the need for penalty calibration; (ii) *Search-space advantage* - by employing the Dicke state within the VQE framework, we drastically reduce the effective search space, restricting the sampling and optimization to the feasible manifold, which improves sample efficiency and convergence stability; and (iii) *Empirical advantage* - in our simulations, the Dicke ansatz combined with CMA-ES achieved higher approximation ratios and more frequent identification of the global optimum than standard ansatzes at comparable parameter counts. We make no claim of asymptotic quantum speedup but demonstrate meaningful structural and practical improvements within the variational paradigm.

This paper is organized as follows: the first section introduces the problem of multiclass portfolio optimization. Subsequently, we provide an overview of VQE and the standard ansatz. We then describe the problem formulation, the Dicke state ansatz, and the methodology employed. Finally, we present the numerical results, empirical findings, and discussions. The paper concludes with a summary of the main contributions and outlines directions for future research in quantum finance.

## Multiclass portfolio optimization

**Fig. 1:**
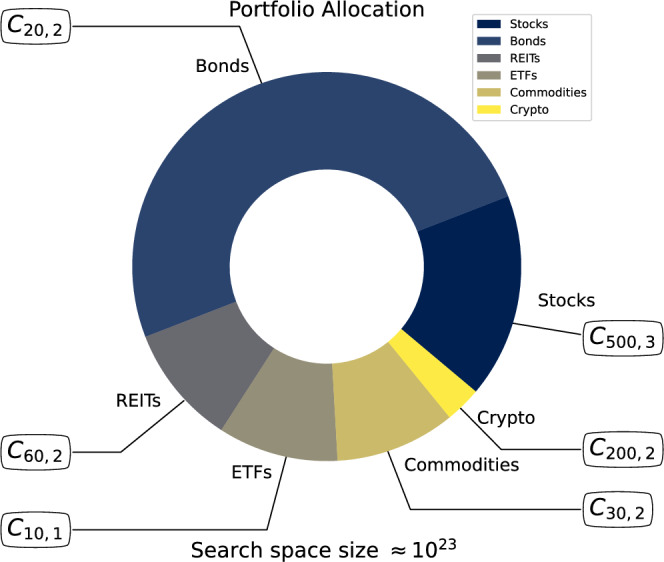
Illustrative example of a multiclass portfolio optimization problem with a predefined asset class allocation to ensure diversification aiming the reduction of market risk. The total number of assets considered is 820, distributed as follows: 500 stocks, 200 cryptocurrencies, 30 commodities, 10 ETFs, 60 REITs and 20 Bonds. For each class there are $C_{n,k}$ possible portfolios, where *n* represents the total number of assets in the class and *k* is the predefined number of assets to be selected. The goal of this portfolio optimization is to find the best set of assets that will produce a portfolio that satisfies the constraints maximizing the return and minimizing the risk.

In practical financial scenarios, optimizing solely for return and risk is insufficient. A realistic and robust portfolio must also incorporate diversification constraints. This involves not only selecting a larger number of assets, but also ensuring representation across different asset classes, such as stocks, bonds, and other financial instruments (see Fig.1).

Diversification reduces exposure to specific market segments, enhances portfolio resilience, and aligns with best practices in risk management. In its classical form, the objective is to select a subset of assets that minimizes portfolio risk while maximizing expected return, under a given level of risk aversion^33^, as formulated below

1 $$\begin{aligned} \min _{x \ \in \ \{0,1\}^n} \ &qx^T\Sigma x - (1-q)x^T\mu + r_f, \nonumber \\ s.t. \ &Ax=b . \end{aligned}$$

where *x* is a binary decision vector such that an entry equal to 1 indicates the inclusion of a corresponding asset in the portfolio, $\Sigma$ is the covariance matrix of asset returns, capturing the portfolio’s overall risk, while $\mu$ represents the expected return vector. The parameter $r_f$ denotes the risk-free rate, such as the return of US Treasury bonds. The matrix $A_{m \times n}$ encodes the linear constraints that govern portfolio selection, where *m* is the number of constraint equations and typically corresponds to the number of asset classes or diversification. In the example of Fig. 1, $a_{ij} = 1$ whenever asset *j* belongs to class *i* and $a_{ij} = 0$ otherwise. To simplify and reduce the number of experimental parameters, we set $q=0.5$.

This formulation enables the inclusion of constraints that promote diversification, by limiting or enforcing the number of assets selected within each predefined class. Such constraints are essential for constructing well-diversified portfolios, which are less exposed to specific sector or asset risks, and thus more resilient to market fluctuations.

## Variational quantum algorithms and ansatz

**Fig. 2:**
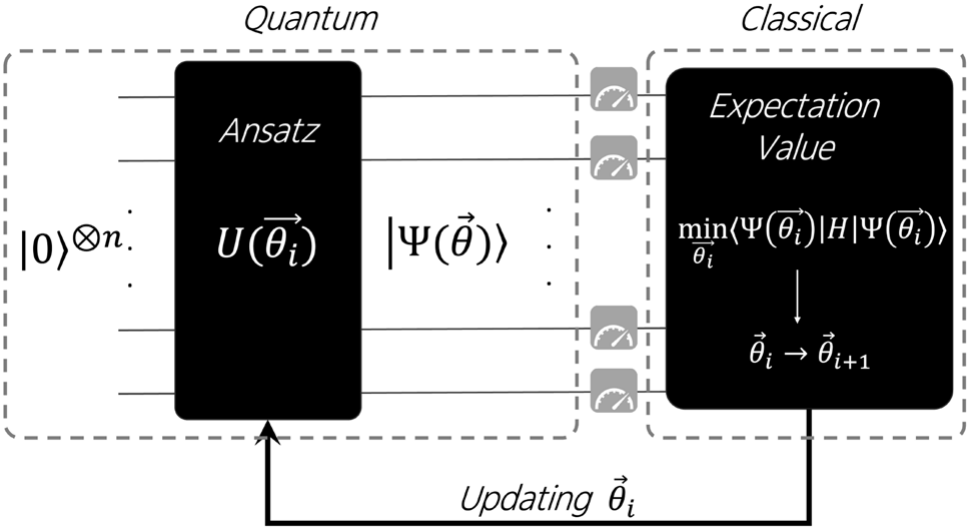
An illustrative example of a VQE routine, which is defined by a quantum state preparation conducted on a quantum device or simulator, followed by an optimization process in a classical computer. In the quantum routine, we start with a quantum state where all qubits are in state $\vert 0\rangle$, this initial state evolves according to the unitary $U(\vec {\theta _i})$, which defines ansatz structure and receives a set of parameters that will define the states probability distribution extracted from a certain number of measurements. The classical routine is focused on updating the set of parameters $\vec {\theta _i}$, in order to minimize the expectation value of the Hamiltonian that encodes the optimization problem. This process is repeated until the maximum number of iterations or when other stopping criteria are achieved.

The primary VQAs for combinatorial optimization are VQE^36^ and the Quantum Approximate Optimization Algorithm (QAOA)^37^. In this work, we focus only on VQE in the context of multiclass portfolio optimization (see Fig.2). Both approaches utilize a parameterized quantum circuit, commonly referred to as an ansatz, together with a classical optimization routine. Their objective is to minimize the expectation value of the problem Hamiltonian (*H*) which encodes the specific combinatorial optimization problem to be solved, as showed in the equation below:

2 $$\begin{aligned} \min _{\vec {\theta } \ \in \ \mathbb {R}^n} \langle \psi (\vec {\theta })\vert H \vert \psi (\vec {\theta })\rangle . \end{aligned}$$

The expectation value defined in equation (2) is lower bounded by the minimum eigenvalue of the Hamiltonian *H*, known as the energy of the ground state $E_0$. The primary goal of this approach is to identify an appropriate ansatz along with an optimal set of parameters $\vec {\theta ^*}$, such that the expected value computed $\langle \psi (\vec {\theta ^*})|H|\psi (\vec {\theta ^*})\rangle$ is equal or close to $E_0$. Quantum mechanics guarantees the existence of this lower bound^38^, although its exact value is generally unknown beforehand. Thus, the variational method provides a practical way to approximate both the ground-state wavefunction and its corresponding energy.

The main difference between VQE and QAOA is that the latter is a special case of the former, once the QAOA ansatz has a well-defined structure based on the adiabatic theorem^38^, which, in general, leads to deeper quantum circuits. There is a trade-off between these algorithms, VQE offers a shallow quantum circuit with a higher number of parameters, meanwhile QAOA offers a deeper ansatz with 2*p* parameters, where *p* represents the number of layers, so in some cases QAOA could solve a problem using fewer parameters but at the cost of using a deeper circuit. In any case, both have the potential to extract useful results from NISQ devices.

**Fig. 3:**
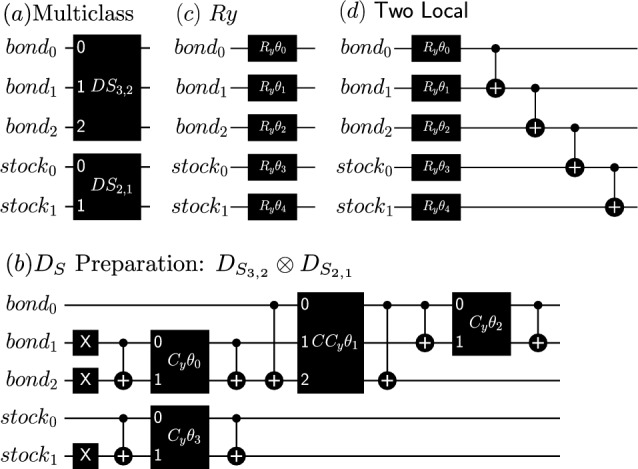
Schematic representation of the ansatzes explored in this work: (**a**) and (**b**) depict the Dicke State, while (**c**) and (**d**) illustrate the $R_y$ and Two Local ansatz, respectively. In this example, the portfolio consists of 5 assets categorized into 2 classes-3 bonds and 2 stocks. The optimization goal is to select 2 bonds and 1 stock to maximize returns and minimize risks. For a consistent comparison, the different ansatzes are configured to have a comparable number of parameters, with the parameterized Dicke state serving as the reference.

Some key challenges in applying VQE to practical scenarios are identifying the optimal ansatz, determining efficient parameter initialization methods, and selecting the most suitable classical optimizer. In this study, we systematically explored various ansatzes to determine the most suitable configuration for our specific problem. The investigated ansatzes can be categorized into three distinct types: (*i*) simple $R_y$ rotation gates, (*ii*) the extensively studied TwoLocal ansatz along with its variants^13^, and (*iii*) the parameterized Dicke state. A detailed schematic representation of each ansatz is provided in Fig.3.

The TwoLocal ansatz is a widely employed variational quantum circuit used in VQE. It consists of two types of parameterized quantum gates arranged in alternating layers: single-qubit rotation gates, such as $R_x$, $R_y$, or $R_z$, and two-qubit entanglement gates, such as *CNOT*. The structure typically begins with rotation gates applied individually to each qubit, followed by layers of entangling gates that couple pairs of qubits, fostering quantum correlations essential for capturing complex solution spaces. This alternating pattern of rotations and entanglement can be repeated multiple times to increase the ansatz’s expressivity. Due to its flexibility and relative simplicity, the two-local ansatz has proven effective in approximating solutions for various optimization problems on current NISQ devices.

## Portfolio optimization and Dicke state

To address portfolio optimization with quantum computing, first we need to convert Eq.(1) to a Quadratic Unconstrained Binary Optimization (QUBO) problem^39^, then the QUBO is converted to an Ising model through the change of variables $x_i = \frac{(1-z_i)}{2}$^40^

3 $$\begin{aligned} \min _{x \ \in \ \{0,1\}^n} qx^T\Sigma x - (1-q)x^T\mu + r_f +\lambda \left( Ax-b\right) ^2. \end{aligned}$$

With that, the equality constraints become a penalty term in the objective function and its intensity is regulated by the Lagrange multiplier $\lambda$. Using information about the structure of the optimization problem, it is possible to encode constraint properties in the ansatz, which produces a quantum state that satisfies the constraints. Therefore, we can remove the penalty term from equation 3 setting $\lambda =0$ if the ansatz guarantees a feasible solution.

**Table 1:** A comparison between different implementations of the Dicke state circuit explored in the literature. The circuits metrics considered are: the complexity of depths of CNOTs, the scaling of number of CNOTs ($n_{CNOTs}$) and number of parameters $n_p$ with *n* and *k*, and topology. These metrics are relevant for resources optimization for Dicke state preparation on the current noisy quantum devices.

Method	Depth CNOTs	$n_{CNOTs}$	$n_p$	Topology
^41^	*O*(*nk*)	$5nk-5k^2$	N/A	all-to-all
Ours	*O*(*nk*)	$5nk-5k^2$	$kn - \frac{k(k+1)}{2}$	all-to-all
^42^	$O(k\log \frac{n}{k})$	*O*(*nk*)	N/A	all-to-all
^42^	$O(k\sqrt{\frac{n}{k}})$	*O*(*nk*)	N/A	grid
^43^	$2(n-k)$	$2nk-3k^2$	$nk-\frac{3k^2}{2}$	LNN
^43^	2*n*	$nk-\frac{k^2}{2}$	$\frac{n(k+1)}{2}-\frac{k^2}{4}$	LNN

To enhance the approach, we can leverage knowledge about the optimization problem structure to design more efficient circuits. For example, let us consider a portfolio optimization problem with *n* variables and constraints that specify the exact number of *k* assets that must be selected to minimize risk and maximize return. In realistic scenarios, usually $k \ll n$ in order to obtain simpler and more explainable portfolios with an adequate level of diversification without reducing potential returns. In this situation, feasible candidate solutions must have a Hamming weight equal to *k*. The size of the set of feasible solutions is given by $C_{n,k} = n!/k!(n-k)!$. Consequently, among all $2^n$ quantum states, we eliminate those that do not meet the specified constraints. By initializing the quantum state in a superposition that encompasses only feasible solutions, the search space is effectively reduced from $O(2^n)$ to $O(n^k)$. Hence, the quantum state suitable for handling such constraints is known as the Dicke state^43^, which is a quantum state related to a fundamental model of quantum optics that describes the interaction between light and matter^44^. The general formula for a uniform distribution of quantum states with *n* qubits and Hamming weight *k* is defined by^45^

4 $$\begin{aligned} \vert D_k^n\rangle = C_{n,k}^{-1/2}\sum _{i}\mathcal {P}_i\vert 0 \rangle ^{\otimes \ (n-k)}\otimes \vert 1 \rangle ^{\otimes \ k}, \end{aligned}$$

where $\mathcal {P}_i$ represents each possible permutation of a quantum state with *n* qubits with *k* qubits equal to $\vert 1 \rangle$. There are several different implementations of the Dicke state (see Table 1). The quantum state defined by Eq.(4), was used as the initial state for QAOA in^46–50^, and in these references the authors tested a variety of mixers. Beyond the scope of optimization, Dicke states are relevant to the following fields: quantum game theory^51^, quantum networks^52^, quantum metrology^53^, quantum error correction^54^ and quantum storage^55^.

Beyond their broad applicability, Dicke states also serve as a foundation for our proposed ansatz in variational quantum algorithms. In this study, we assume all-to-all qubit connectivity to emphasize the conceptual contribution of the Dicke-state formulation and its ability to enforce diversification constraints without penalty terms. Nevertheless, the approach is not limited to this topology. As shown in Table 1, existing Dicke-state preparation circuits support all-to-all, grid, and linear-nearest-neighbor (LNN) architectures, with different trade-offs in depth and two-qubit gate count. Hence, the framework is *hardware-agnostic*, as preparing each class subspace only requires initializing a fixed-Hamming-weight superposition.

This ansatz fits perfectly with the portfolio optimization problem subject to a constraint of a fixed number of products, since it creates a superposition in the space of feasible solutions. To address portfolio optimization through VQE with the Dicke state ansatz, we use its parameterized version (see Fig.3b):

5 $$\begin{aligned} \vert D_k^n (\vec {\theta })\rangle = \sum _{i}\mathcal {P}_i a_i(\vec {\theta })\vert 0 \rangle ^{\otimes \ (n-k)}\otimes \vert 1 \rangle ^{\otimes \ k}, \end{aligned}$$

where $a_i(\vec {\theta })$ represents amplitude probability as a function of the parameters $\vec {\theta }$. A similar approach was presented by^43^, which focused solely on the preparation of a single Dicke state, thus mimicking portfolio optimization without diversification constraints. In this paper, we use the implementation given by^41^, creating a non-uniform Dicke state parameterizing the circuit implementation and incorporating the multiclass optimization by including multiple Dicke states representing the multiple classes. Each Dicke state represents a class with a set of products (number of qubits *n*) and will encode the constraints of the exact number of assets which will be selected by class (parameter *k*) (see Fig.3a). The equation below dictates the number of parameters $n_p$ of a Dicke state ansatz

6 $$\begin{aligned} n_{p} = \sum _{i=1}^{m}k_in_i - \frac{k_i(k_i+1)}{2} \end{aligned}$$

where $k_i$ is the number of states $\vert 1 \rangle$, that corresponds to the Hamming weight associated with the budget constraint. Equation (6) was derived empirically, for further details see Suplementary Material.

For $m=1$, we have a unique Dicke state ansatz with *n* qubits and Hamming weight *k*, but $m>1$ implies a tensor product of different Dicke states. The summation is over the number of classes represented by the number of Dicke states in the tensor product of the initialization.

## Results

We addressed the portfolio optimization problem using the SCIP optimizer, treating its solution as the benchmark reference (see Supplementary Material). The running time was determined by averaging the results from 100 executions. This average running time served as the basis for comparing the performance of classical methods with hybrid VQE routines. However, it is important to note that we did not expect that the VQE approach would outperform the classical methods in terms of speed.

The experiments were performed in three different scenarios as described in Table 2. For Scenario I, we ran the VQE algorithm for 20 different ansatzes (Dicke state, $R_y$, and 18 variations of Two Local). For Scenarios II and III only the Dicke state was used. For all scenarios, we tested 5 classical optimizers with 1000 iterations, 100 randomly sampled initial points (ansatz parameters), 4096 shots per circuit, totalizing more than 60 billion executions ($\approx n_{ansatz}\times n_{optimizers} \times n_{executions} \times n_{shots} \times n_{iterations}$). More details of the variations of the two-local ansatz used here can be found in Supplementary Material. All data used in this work are publicly available and were obtained using the Yahoo Finance API.

**Fig. 4:**
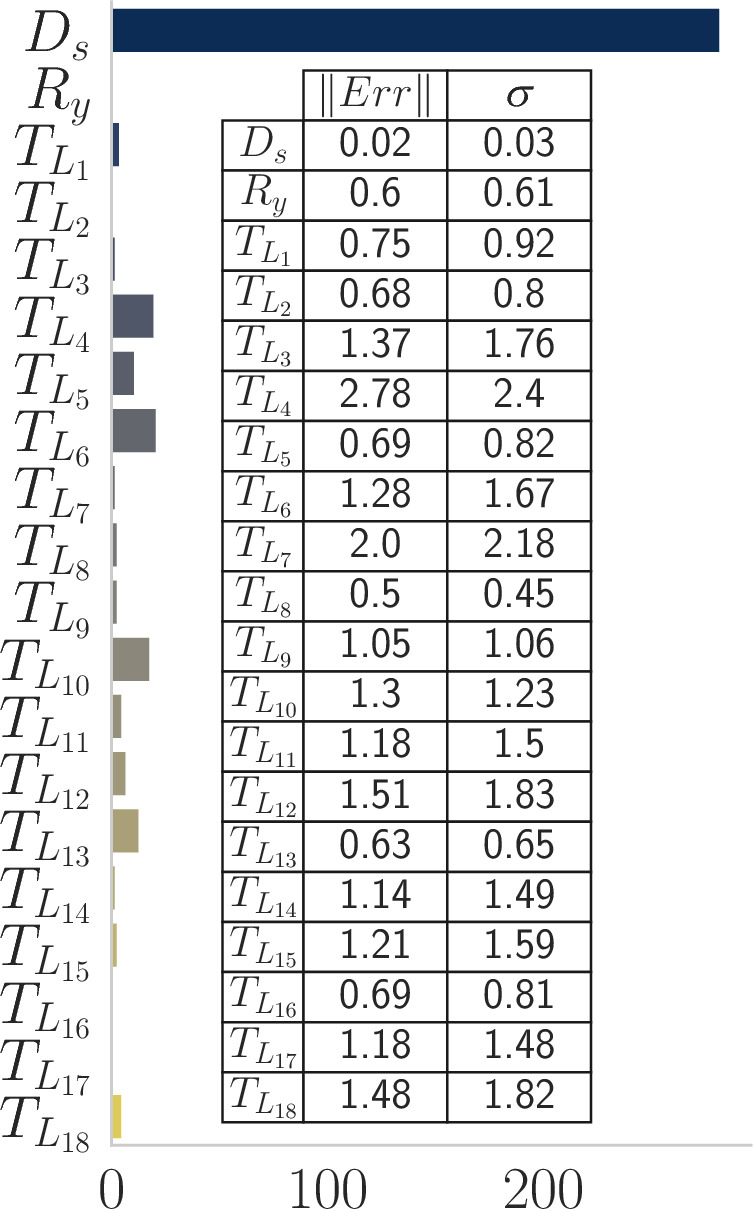
Ansatz comparison results for Scenario I taking into account all optimizers. The histogram shows the number of trials where each ansatz found the optimal solution as its most common output. Notably, out of the 20 ansatzes tested, the Dicke state demonstrated the best performance, independently of the classical optimizer. Out of 500 runs, in more than $50\%$, the VQE-Dicke state found the optimal result as the one with the highest probability. The inside table shows the absolute error $(\vert \vert Err\vert \vert )$ and the standard deviation $(\sigma )$ between the expected value of the quantum solution and the target. Again, the VQE-Dicke state presented the best metrics.

We employ Scenario I to identify the most effective ansatz for multiclass portfolio optimization. Among the 500 trials conducted per ansatz, the parametrized Dicke state emerged as the best performing approach, regardless of the classical optimizer (see Fig.4). For this initial evaluation, we only verified that the optimal solution consistently emerged as the state with the highest probability without considering the magnitude of this probability. It is evident that the Dicke state has been the most effective ansatz so far. Because of that, we only employ it for the other scenarios to assess its performance and evaluate the impact of classical optimizers.

From a theoretical standpoint, the improved optimization performance of the Dicke-state ansatz arises from its structural design, which confines the variational search to the feasible subspace defined by the diversification constraints. By preparing the quantum state as a tensor product of Dicke states, the search space is reduced from the full $2^n$ Hilbert space to $\prod _i C_{n_i,k_i}$ a substantial reduction in realistic scenarios where $k_i \ll n_i$. This restriction removes infeasible configurations, concentrates the optimization on meaningful portfolio states, and preserves the symmetry of fixed Hamming weight. Consequently, the optimizer operates on a smoother energy landscape, improving convergence stability and accuracy relative to more general ansatzes.

**Table 2:** The table shows the different scenarios we use to evaluate the performance of parametrized Dicke state in the context of multiclass portfolio optimization. Where $n_a$ represents the number of assets (that define the number of qubits used in the scenario), $n_c$ number of classes, $n_s$ amount of select assets, $n_p$ number of parameters of Dicke state ansatz. $\vert D_k^n\rangle$ is a superposition of all states with *n* qubits and *k* qubits equal $\vert 1\rangle$. Each state represents a class where *n* is the total number of assets available to choose and *k* is the number of assets we must select from it. The parameter $\vec {\theta }$ was omitted in the state for better visualization. The last column shows the actual size of search space for each scenario.

Scenario	$n_{a}$	$n_{c}$	$n_{s}$	Ansatz	$n_{p}$	$n_{\text {search space}}$
I	10	1	4	$\vert D_4^{10}\rangle$	30	210
II	25	5	5	$\vert D_1^{5}\rangle ^{\otimes \ 5}$	20	3125
III	25	5	9	$\vert D_2^{5}\rangle ^{\otimes \ 2}\vert D_1^{5}\rangle ^{\otimes 2}\vert D_3^{5}\rangle$	31	25000

**Fig. 5:**
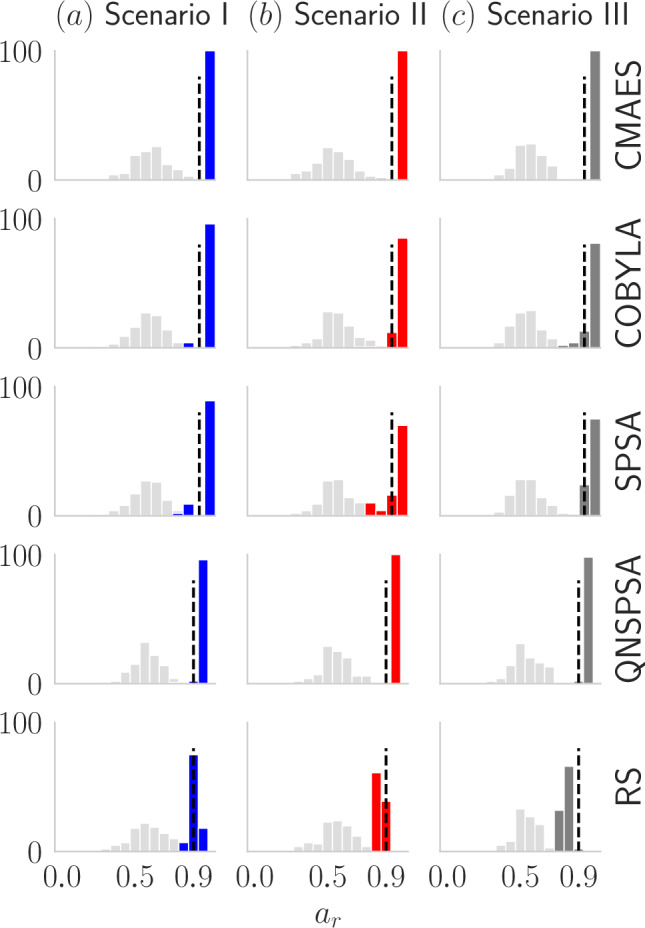
Plots (**a**), (**b**), and (**c**) compare the approximation ratio ($a_r$) distributions before (gray) and after (colored) optimization for each scenario (columns) and optimizers (rows). The gray represent $a_r$ distributions with the initial parameters, while the colored ones reflect optimized parameters, based on 100 experiments per optimizer. A vertical black dashed line marks $a_r = 0.9$. Across all optimizers and scenarios, the initial distributions shift toward $a_r \ge 0.9$, indicating the positive impact of the hybrid approach and optimizers. In Scenario I (blue) all the optimizers guide the solution to the optimal region.

**Fig. 6:**
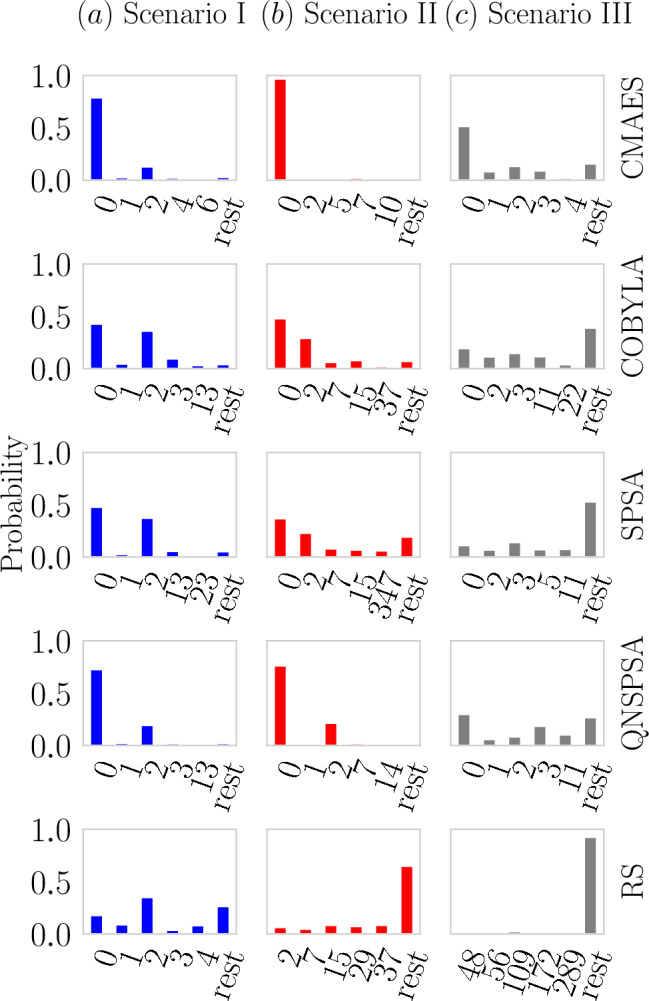
Plots (**a**), (**b**), and (**c**) display the sampling probabilities of each bit string, ordered from lower to higher energy, aggregated over 100 runs. The target state is positioned at 0 based on this energy ordering. We present the five best results from each optimizer across all scenarios, with the tick rest aggregating outcomes outside the top five. Overall, CMA-ES and QNSPSA achieved the best performance, as they concentrate probability distribution in the optimal region and assign the highest probability to the ground state.

Another challenge in the evaluation of hybrid algorithms is to measure the effect of the classical optimizer on the solution. As mentioned before, we tested five different optimizers: CMA-ES^56^, COBYLA^57^, Random Sampler, SPSA^58^ and QNSPSA^59^. The evaluation takes into account three different metrics: the approximation ratio, the frequency with which the right answer appears in the 100 trials per optimizer, and the quality of the quantum output for each trial (the probability of measuring the target state). The approximation ratio is defined as^23,60–62^

7 $$\begin{aligned} a_r = \frac{\left( E_{max}-\langle H \rangle _{\psi (\vec {\theta ^*)}}\right) }{(E_{max}-E_0)}, \end{aligned}$$

where $\langle H \rangle _{\psi (\vec {\theta ^*)}}$ is the expected value of the problem Hamiltonian computed with the ansatz and the set of parameters obtained at the end of optimization. $E_0$ is the ground-state energy associated with the optimal solution that was calculated by computing the lowest eigenvalue for the problem Hamiltonian. $E_{max}$ represents the highest Hamiltonian eigenvalue. The metric above measures how close or distant the solution is from the optimal. For example, an approximation ratio equal to 1 means that the solution is equal to the optimal one.

When comparing the results of the experiments in different scenarios, it is clear that CMA-ES emerged as the optimizer that exhibited the highest frequency of finding the target state with the highest probability among all the 100 trials (see Table 3). In terms of time, COBYLA was the fastest optimizer, followed by CMA-ES in second place. QNSPSA, the second solver that demonstrated good performance in finding the global optimal, was surprisingly costly in terms of time, resulting in the worst performance among all optimizers. This result indicates the potential of using CMA-ES as a good choice for hybrid algorithms. More explorations must be done, but, at least for these experiments, CMA-ES surpasses the other optimizers.

**Table 3:** A summary of experiments results for each scenario considering only Dicke state ansatz. The column *All* represents the frequency of the optimal global solution $(x^*)$, among 100 experiments, counted if it appears with highest probability. $p(x^*) \ge 0.95$ filter only the quantum outputs which the probability of the optimal state is greater than 0.95. As can be seen, by applying this filter the frequency in CMAES drastically reduces in some scenarios (numbers indicated in Bold and Italic). The last column represents the average running time and the standard deviation of each optimizer.

Scenario	Optimizer	All	$p(x^*) \ge 0.95$	Time (s)
I	CMA-ES	$\boldsymbol{88}$	***25***	$82.8 \pm 0.6$
	COBYLA	44	32	$29 \pm 4$
	QNSPSA	74	$\boldsymbol{65}$	$601 \pm 3$
	RS	27	0	$82.5 \pm 0.7$
	SPSA	49	44	$154.5 \pm 0.8$
II	CMA-ES	$\boldsymbol{98}$	$\boldsymbol{97}$	$181 \pm 4$
	COBYLA	49	35	$43 \pm 6$
	QNSPSA	76	73	$981 \pm 9$
	RS	6	0	$202 \pm 1$
	SPSA	40	25	$302 \pm 6$
III	CMA-ES	$\boldsymbol{70}$	***1***	$335 \pm 12$
	COBYLA	21	5	$119 \pm 21$
	QNSPSA	30	$\boldsymbol{27}$	$1541 \pm 20$
	RS	0	0	$376 \pm 3$
	SPSA	12	4	$467 \pm 17$

It is clear that the optimizers guide the distribution toward the right direction, this fact can be seen in Fig.5, where we compare the approximation ratio distribution before and after the parameter optimization process. Note that the values of the initial distribution are below $a_r=0.9$, but after optimization we can see a displacement to the right, which means that optimization succeeded in obtaining a set of parameters which generates a quantum state whose state distribution has a high probability of measuring a state with a high approximation ratio.

Note that even when the probability of measuring the target state is low (see probability of state of measure 0 in Fig. 6), $a_r \gtrsim 0.9$ for most optimizers, see Fig. 5 (colored bars). This phenomenon is linked to Eq.(7), which, from a quantum perspective, acts as a weighted average representing the expected value of the Hamiltonian. This implies that the approximation ratio of the quantum output distribution is essentially a weighted sum of the approximation ratios of individual states. As a result, when the system moves toward the optimal region post-optimization, where most states in the distribution have lower energy, the approximation ratio generally becomes higher.

The clarity of the results is enhanced upon examining Fig.6. After conducting 100 experimental runs, we aggregated the data and analyzed the frequency of each bit string, arranging them in ascending order of energy. For improved visualization, we retained only the top five bit strings, consolidating all others into a *rest* category. It is evident that CMA-ES consistently exhibits the highest probability of sampling the optimal solution in all scenarios and dominates the top five regions. QNSPSA, COBYLA, and SPSA show comparable behaviors, ranking second. Although they are capable of achieving the optimal solution, in instances where they do not, they frequently yield solutions that are close to the global optimum.

**Fig. 7:**
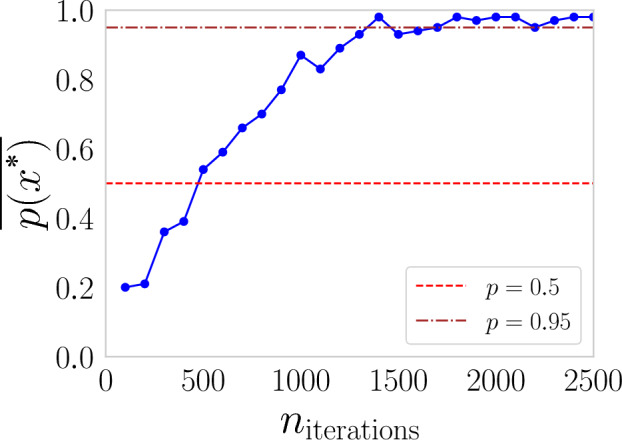
The result expresses how the probability to achieve the target state evolve as we increase the number of iterations in CMA-ES optimizer in scenario I. Each point in the curve represents 100 executions of VQE-Dicke with 4096 shots for a fixed number of iterations of CMA-ES. Note that each execution initializes a random set of parameters for VQE. After each execution we extracted the probability of measuring the target state and compute the average among all 100 runs. As the number of iterations increases, independently of the parameters initialization, $\overline{p(x^*)}$ also increases. When $n_{\textrm{iterations}} \gtrsim 1500$, the points fluctuate around 0.95, indicating convergence to the region where $p(x^*) \gtrsim 0.95$. These residual fluctuations arise from stochastic optimizer initializations and finite-shot sampling, and occasional dips below 0.95 are therefore expected and do not compromise the convergence trend.

Despite the high performance of CMA-ES in all scenarios, the quality of the quantum output is not consistent. To investigate why $p(x^*)$ is widely distributed, we evaluated the impact of the number of iterations in $p(x^*)$. As can be seen in Fig.7 as the number of iterations increases, the average probability of finding the target state also increases, achieving a more accurate result. The experiment was performed in scenario I and with $n_{interations} \gtrsim 1500$ the solution reaches the optimal region $(p(x^*) \ge 0.95)$. The result strongly suggests that, in addition to the capacity to find the target state, it is possible to improve the quality of the quantum output by increasing the number of iterations.

It is important to mention that recent studies^61,63,64^ suggest the potential quantum advantage of variational algorithms like VQE and QAOA arises when the number of function evaluations $(n_{calls} \equiv n_{shots} \times n_{iterations})$ remains smaller than the size of the effective search space ($n_{\text {search space}}$). By applying the Dicke state ansatz in our problem, we significantly reduce the search space from $2^n$ by $\prod _{i}^{n_{c}} C_{n_i, k_i} \sim O(n^k)$. However, for the set of experiments provided here, $n_{calls} \sim 4.096.000 \gg n_{\text {search space}}$ as can be seen in the last column of Table 2.

## Conclusion

In this work, we introduce an innovative approach to exploring multiclass portfolio optimization through a parameterized version of multiple Dicke states within a VQE framework. We analyze three distinct scenarios by varying the complexity of the search space and the number of parameters in the parameterized circuit. Our comparative evaluation indicates that Dicke states outperform other ansatzes, making them a suitable choice for this type of problem.

Furthermore, we examine the impact of various optimizers within this hybrid algorithm. Our results indicate that CMA-ES outperforms other optimizers in both execution time and convergence to the optimal solution. However, achieving higher-quality quantum outputs requires a larger number of iterations and tuning of the optimizer parameters. Additionally, all optimizers tested here appear to find parameters that guide the quantum distribution output toward regions close to the ground state. The Random Sampler was shown to be the worst optimizer.

Another intriguing aspect we began to evaluate is the relationship between state fidelity and optimal parameters, as discussed in the Supplementary Material. In future research, our aim is to address the open questions highlighted here and to further investigate the use of the QAOA with Dicke states for multiclass portfolio optimization. This will include exploring different mixer Hamiltonians.

All our results were obtained through simulations, as our primary objective was to gain a deeper understanding of the algorithm’s potential for solving realistic portfolio optimization problems, encoding the diversification constraints in the quantum state preparation.

Although the proposed Dicke-state framework achieved promising results, further investigation is needed to evaluate its scalability and practical benefits in large-scale, real-world portfolio optimization. The potential advantage of variational algorithms emerges when the number of function evaluations remains smaller than the effective search space, motivating future studies combining the Dicke ansatz with CMA-ES and Conditional Value at Risk (CVaR) to explore possible regimes of quantum advantage. While current simulations assume all-to-all connectivity, the approach is hardware-agnostic and compatible with realistic 2D or sparse architectures, offering structural efficiency by reducing the feasible subspace and eliminating penalty terms. Preliminary hardware tests on IBM quantum devices can be seen in the Supplementary Material and confirm the expected sensitivity to noise and circuit depth, reinforcing the focus on noiseless simulations to isolate algorithmic behavior. Finally, quantum-optimized portfolios showed consistency with classical allocations in risk–return balance and diversification quality, encouraging future work on explicit financial performance metrics.

Future research is also needed to test these findings on current quantum hardware, comparing the effectiveness of Zero-Noise Extrapolation (ZNE), Probabilistic Error cancellation (PEC) and CVaR in mitigating errors during the calculation of expectation values. Our goal was not to provide definitive proof of this impact, but rather to test the viability of utilizing VQE in a more realistic financial scenario and open a new direction of exploration that is encoding optimization constraints in state preparation. We believe that our findings represent a significant step towards this objective.

## Supplementary Information

Supplementary Information.

## Data Availability

The datasets generated and/or analyzed during the current study are available in the Yahoo Finance and can be downloaded using the following python library: yfinance. The period of the datasets considered in this work are: 2023-12-18 until 2024 12-16 and 2024-01-02 until 2025-01-02.

## Code availability

The underlying code for this study is not publicly available but may be made available to qualified researchers on reasonable request from the corresponding author.
