# Supplementary material for: Multiclass portfolio optimization via variational quantum Eigensolver with Dicke state ansatz
Source: Sci Rep. 2026 Feb 13;16:6208. doi: 10.1038/s41598-026-36333-4 (PMC12905263; doi:10.1038/s41598-026-36333-4)
Supplement: Supplementary file 1 — Supplementary Information. [file 41598_2026_36333_MOESM1_ESM.pdf]

Supplementary material for "Multiclass Portfolio Optimization via Variational Quantum Eigensolver with Dicke State Ansatz"

SUPPLEMENTARY METHODS

I. EXPERIMENT SETTINGS

The VQE algorithm simulations were performed using the instance ml.g5.xlarge on Amazon SageMaker. This machine has four virtual CPUs, 16 GB RAM and NVIDIA GPU A10G with 24 GB GDDR6 VRAM. This system runs on Linux, with Python version 3.9.5, CUDA version 12.4, NVIDIA Graphics driver version 550.144.03. The Python libraries used to perform the simulations are: qiskit version 1.3.2 [1], qiskit-aer-gpu version 0.15.1, qiskit-algorithms version 0.3.1, qiskit-optimization version 0.6.1, numpy version 2.0.2, optuna version 4.1.0 and CMA-ES version 0.11.1. All initial points were generated with NumPy random methods using the seed 42, with the exception for CMA-ES and Random Sampler due to optuna's parameter sampling method. However, we guarantee that all parameters are in the interval $[0, 2\pi]$, independently of the method used to sample the parameters values. CMA-ES and Random Sampler were used through the Optuna interface, whereas COBYLA, SPSA and QNSPSA were used through the Qiskit algorithms package.

As mentioned in the main paper, we solved each scenario proposed with the classical optimizer SCIP and considered its results as a reference. These results are summarized in Table I.

Scenario	Objective function value	Running time (s)
I	-0.818106	0.01 $\pm$ 0.001
II	-1.474237	0.03 $\pm$ 0.001
III	-2.00332	0.03 $\pm$ 0.001

TABLE I. A summary of the reference values computed using SCIP optimizer for each scenario. The column Objective function value holds the optimal values for each case. The column Running time represents the mean time spent and the standard deviation of 100 runs of the optimization problem.

II. TWOLOCAL ANSATZ

The TwoLocal circuit is an ansatz that alternates rotation and entanglement layers. The rotation gates are applied in each qubit individually and the gates RX, RY, RZ, U_1, U_2 and U_3 are some examples of rotation gates that we can use in this ansatz. Meanwhile, the entanglement gates are used to create entanglement between pairs of qubits considered in each entanglement structure design, for instance: we can create entanglement between all qubits or just in some qubits in a way that connects the qubits through a line. In order to create the desired entanglement structure, we can use gates such as: CX, CY, CZ, CRX, CRY and CRZ. For instance, in Table II we show some TwoLocal settings used in Scenario I experiments.

III. DERIVATION OF THE NUMBER OF PARAMETERS IN THE PARAMETRIZED DICKE STATE

The total number of parameters is directly related to the number of gates CRY and $CCRY$, and each of these gates has one free parameter. We defined a range of values for n and considered $[1, n-1]$ as the range for k. Then, we constructed the Dicke state ansatz for all values of n and k defined in the ranges mentioned above and computed the total number of parameterized gates. With these data in our hands, we were able to plot the curves (see Figure 1) and also perform a regression to derive a formula that dictates the relationship between the number of qubits and the Hamming weight, with the number of parameters in the Dicke state ansatz.

Taking into account a fixed value for k and varying the number of qubits n in the range $[k+1, d]$, where $d > k+1$, and building the Dicke state ansatz circuit for each value of n, we can calculate the number of parameters. Plotting

Name	Qubits	Parameters	Rotation Blocks	Entanglement Blocks	Entanglement	Repetitions	Skip Final Rotation Layer
TwoLocal1	10	30	3 x RY	CX	Full	1	True
TwoLocal2	10	30	1 x RY	CX	Full	2	True
TwoLocal3	10	30	1 x RY	CX	Full	3	False
TwoLocal4	10	30	3 x RY	CX	Linear	1	True
TwoLocal5	10	30	1 x RY	CX	Linear	2	True
TwoLocal6	10	30	1 x RY	CX	Linear	3	False
TwoLocal7	10	30	3 x RY	CX	Reverse Linear	1	True
TwoLocal8	10	30	1 x RY	CX	Reverse Linear	2	True
TwoLocal9	10	30	1 x RY	CX	Reverse Linear	3	False
TwoLocal10	10	30	3 x RY	CX	Circular	1	True
TwoLocal11	10	30	1 x RY	CX	Circular	2	True
TwoLocal12	10	30	1 x RY	CX	Circular	3	False
TwoLocal13	10	30	3 x RY	CX	SCA	1	True
TwoLocal14	10	30	1 x RY	CX	SCA	2	True
TwoLocal15	10	30	1 x RY	CX	SCA	3	False
TwoLocal16	10	30	3 x RY	CX	Pairwise	1	True
TwoLocal17	10	30	1 x RY	CX	Pairwise	2	True
TwoLocal18	10	30	1 x RY	CX	Pairwise	3	False

TABLE II. Settings used to compose each TwoLocal variant consired in the experiments in Scenario I. All circuits for Scenario I were created to have 30 parameters and 10 qubits, then we just varied the amount of rotation blocks, entanglement structure, the number of layers repetitions and whether we skip the final rotation layer. In this case we only used RY and CX gates because relative phases are not relevant.

n_p by n, we saw a polynomial function of degree 1, then we can write the following expression

$$n_p = mn + b. \tag{1}$$

The angular coefficient can be calculated by the following equation

$$m = \frac{n_p^f - n_p^i}{n_f - n_i}, \tag{2}$$

computing the value of m with the data obtained from the quantum circuits of Dicke state ansatz, we found that $m = k$, which implies that the angular coefficient is equal to the Hamming weight of the Dicke state. The linear coefficient can be computed using the following expression

$$b = n_p^i - kn_i, \tag{3}$$

and in order to find out if there is a general rule for b, we tested different values of k for a fixed d and we found that the value of the linear coefficient is given by

$$b = \frac{k(k+1)}{2}. \tag{4}$$

Thus, combining all the elements computed above, we obtain the following equation for a Dicke state ansatz with n qubits and Hamming weight k

$$n_p = kn - \frac{k(k+1)}{2}. \tag{5}$$

IV. DICKE STATE ANSATZ ON NOISY DEVICES

Once a good ansatz has been identified, it is important to improve its implementation in hardware to improve the results. In reference [2], the authors proposed a Dicke state ansatz version that is more efficient to run in the

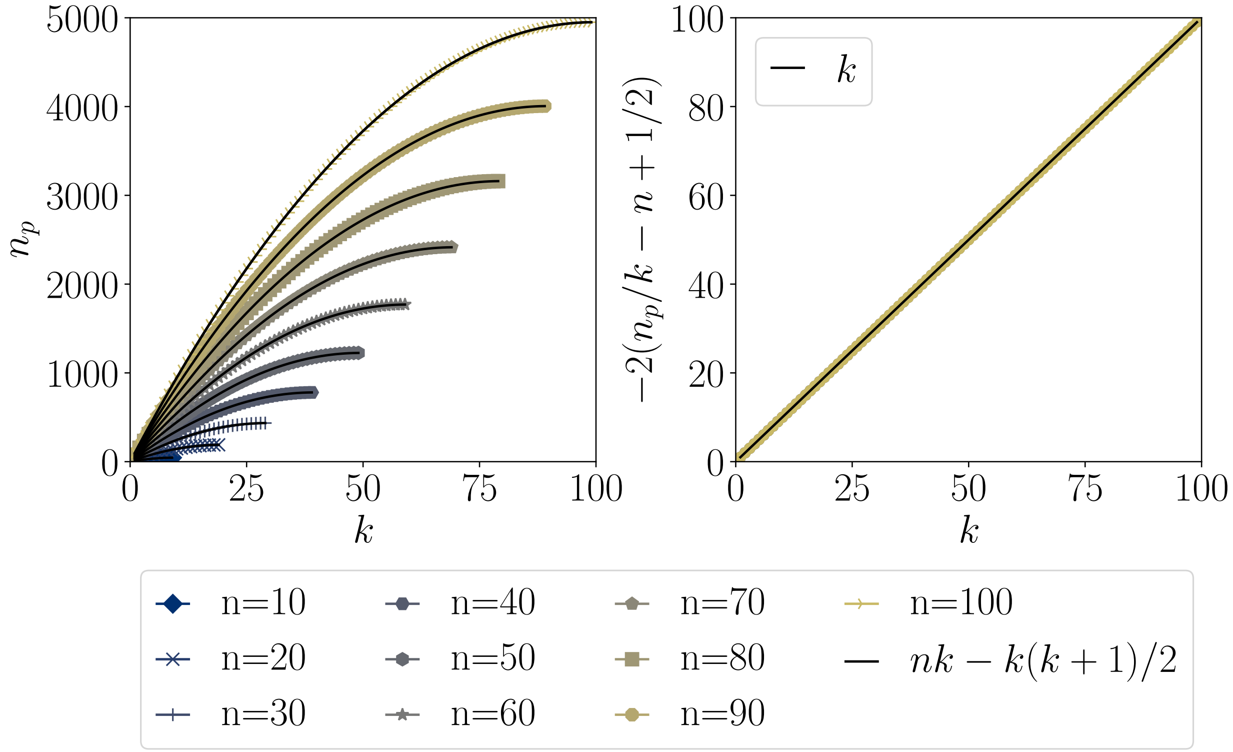

SUPPLEMENTARY FIGURE 1. Scaling of the number of parameters in Dicke state ansatz for different numbers of qubits and varying the Hamming weight k. Note that for $k=0$ and $k=n$ we don't need a parameterized Dicke state, because $k=0$ is equivalent to $|0\rangle^{\otimes n}$ and $k=n$ is the state $|1\rangle^{\otimes n}$, then the range applied to k is $[1, n-1]$. The curves defined by different values of n and k can be unified by the expression $nk-k(k+1)/2$, as depicted in the plot in the right.

current noisy quantum hardware, reducing the number of entangling gates and also considering qubits connections constraints. Another way to improve the depth of the Dicke state circuit proposed in [2, 3], it is the parallelization of entangling gates, with which we can optimize the circuit depth in order to create quantum circuits which are suitable for the current quantum hardware coherence time. A topic to highlight is the Dicke state sensitivity to errors of two types: bit-flip and readout errors. For instance, we can measure the impact of bit-flips, in our ansatz, modeling this type of error as described below and manipulating the probability p of occurrence of a bit-flip [4]

$$\rho = (1-p)\rho_0 + p\sigma_x\rho_0\sigma_x, \tag{6}$$

where ρ_0 represents the density matrix related to the Dicke state ansatz. Then we can use the noisy state ρ to calculate the Hamiltonian expected value through [5]

$$\langle H \rangle = \mathrm{tr}(\rho H), \tag{7}$$

and with that we will be able to estimate the impact on our objective function. Both errors can destroy the main characteristics of this quantum state, that is, a constant Hamming weight, because depending of the number of bit-flips and in which qubits they will occur, this kind of error can increase or reduce the Hamming weight, which will reflect in adding Hamiltonian terms that will have a great impact on Hamiltonian expectation value. Then, it is expected that the results obtained from current noisy quantum hardware will not achieve the same level of convergence that we achieved with noiseless simulation, because bit-flips and readout errors can make parameter optimization more challenging, since noise can also induce Barren Plateaus [6].

One last type of error that can cause issues during parameter optimization is the coherent error, more specifically, an error of the form $U(\theta+\delta\theta)$, where $\delta\theta$ represents a displacement in the original parameter. In order to mitigate the damage that the errors described above can cause, we can use some error mitigation techniques. For example, to mitigate errors in expectation values we can use ZNE [7–9], PEC [10, 11] and Probabilistic Error Amplification

(PEA) [12, 13]. For readout errors, we can apply statistical corrections [14, 15], M3 technique [14] or Twirled Readout Error Extinction (T-REX) [15]. The usage of the error mitigation techniques mentioned above, combined with advanced transpiling methods are highly recommended, in order to produce optimized quantum circuits to current hardware, improving the chances of obtaining good results. Another interesting direction in which we would like to explore CVaR as the objective function [16, 17], once CVaR seems to be a noise-resistant loss function for variational quantum algorithms.

In order to assess the effectiveness of our simulation results on current quantum hardware, we carried out experiments utilizing the IBM QPU *ibm_pittsburgh*. These tests were executed in Scenario I, employing the most optimal set of parameters post-optimization. The parameters we identified ensured a 100% probability of sampling the global optimum. We analyze the distribution obtained from the hardware against the simulation, which in this scenario matches the ideal result. As observed in Supplementary Figure 2, the hardware results are quite noisy and do not identify the global optima as the bit string with the highest sampling probability. Additionally, it is noteworthy that the Hamming weight of the sampled bit strings failed to maintain the Hamming established by $k = 4$, since infeasible solutions appear in the results. This result aligns with the discussion in this section, where it was emphasized that in NISQ hardware, infeasible solutions may arise due to bit-flip and readout errors.

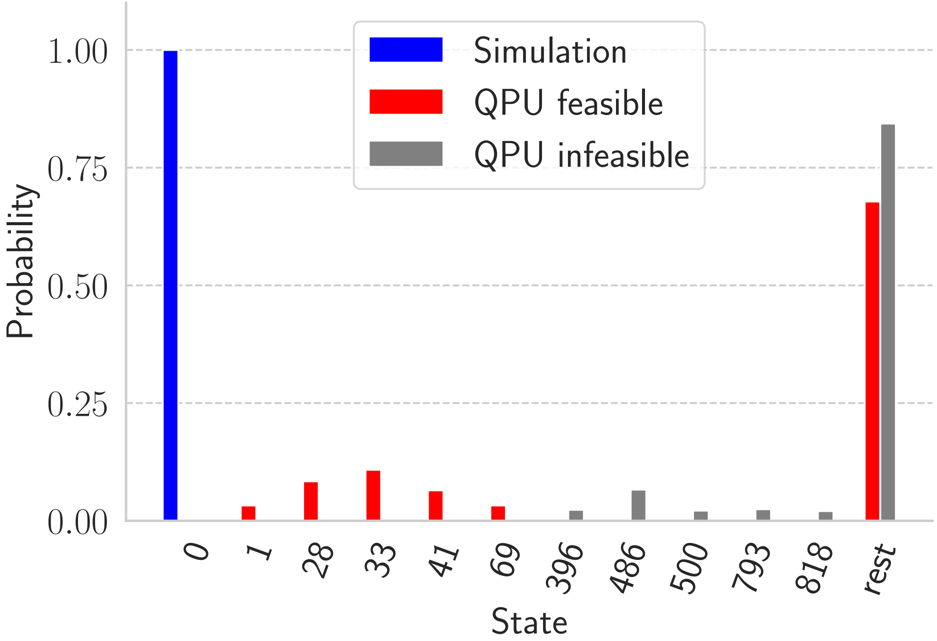

SUPPLEMENTARY FIGURE 2. Scenario I: A comparison between a simulation using optimal parameters that achieves the ground state with a 100% probability and the distribution obtained from hardware using the identical circuit and parameters. The states are labeled with integer numbers according to their energy levels. Thus, the ground state is labeled as 0, the first excited state as 1, and this pattern continues similarly. In blue, we have the simulation results, while the hardware results are in red and gray. The red indicates the feasible bit strings, whereas the gray the infeasible bit strings, which do not comply with the Hamming weight constraints. Observe that the search space size is 210, meaning that indexes greater than this indicate infeasible bit strings. The optimization level used in Qiskit Transpiler was 3.

Additionally, we calculate the expectation value of the Ising Hamiltonian for Scenario I using the Qiskit Estimator primitive over 100 executions on IBM quantum hardware. We chose the set of parameters that yielded the best outcome in simulations and incorporated these parameters into the Dicke state ansatz. Subsequently, the expectation value was calculated 100 times using the same quantum circuit. The results are shown in Supplementary Figure 3

It is important to note that these experiments were performed using the default settings of Qiskit Sampler and Estimator, and we also considered the standard transpilation techniques adopted in Qiskit transpiler. The results presented above show what we expected to see in noisy hardware without circuit optimizations and error mitigation techniques, however these data do not contradict our simulation results, they reflect the challenge that is extract a result that is comparable with a noiseless simulation. In future research, we would like to address these problems of hardware performance with a more optimized circuit and also using the error mitigation techniques mentioned.

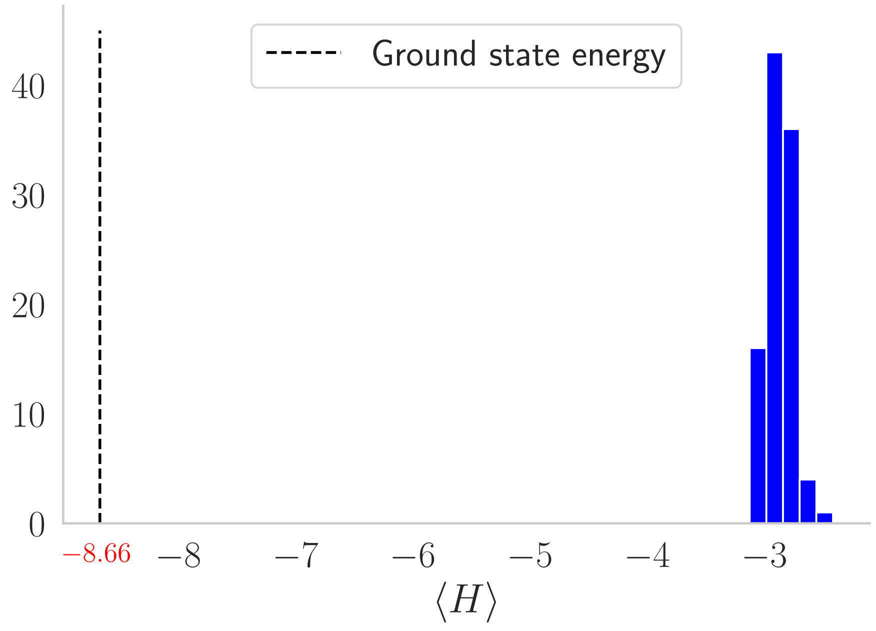

SUPPLEMENTARY FIGURE 3. Calculation of the expected value of the Hamiltonian using 100 Qiskit Estimator executions in Scenario I. The vertical dashed black line indicates the ground state energy that we aim to reach. It is evident that the distribution is significantly distant from the optimal regime.

V. QUANTUM STATE FIDELITY ANALYSIS

An additional analysis that we performed was on the quantum state fidelity between states at the end of the optimization process, but also on their state fidelity with reference state. Here we only consider results obtained with Dicke state ansatz simulations using CMA-ES as optimizer, these results are summarized in Figure 4.

The state fidelity computation was done considering pure states, then we used the following mathematical expression [4]

$$F(|\psi(\vec{\theta}_i^*)\rangle, |\psi(\vec{\theta}_j^*)\rangle) = |\langle\psi(\vec{\theta}_j^*)|\psi(\vec{\theta}_i^*)\rangle|^2, \tag{8}$$

where $\vec{\theta}_i^*$ and $\vec{\theta}_j^*$ represent two different optimal set of parameters for the states described in Table **??**. To compute the state fidelity, we consider a parameterized quantum circuit that is used to compute the kernel in Quantum Support Vector Machine (QSVM) [18], where we apply an unitary operator $U(\vec{\theta})$ that prepares the desired state and its adjoint with different set of parameters, as stated in the following expression

$$|\Phi\rangle = U^{\dagger}(\vec{\theta}_j)U(\vec{\theta}_i)|0\rangle^{\otimes n}. \tag{9}$$

Then, our estimation of fidelity will be equal to the probability of measuring $|0\rangle^{\otimes n}$. This fidelity estimation could also be done with the SWAP test [19].

Our finding here was that these different sets of optimal parameters lead to quantum states that have a high fidelity to the reference state. We also noted a certain level of similarity between these quantum states, as shown by the heat maps plots in the Supplementary Figure 4. However, we cannot extract useful information from the distance between the parameter sets, because it is not clear how we can define what is close or distant in the parameter space.

Perhaps, with this information in hand, we can extract some useful information about the optimization landscape to improve the success of this method. A future research line would design a neural network to provide a good initial starting set of parameters considering this information about the similarity between the states that have a

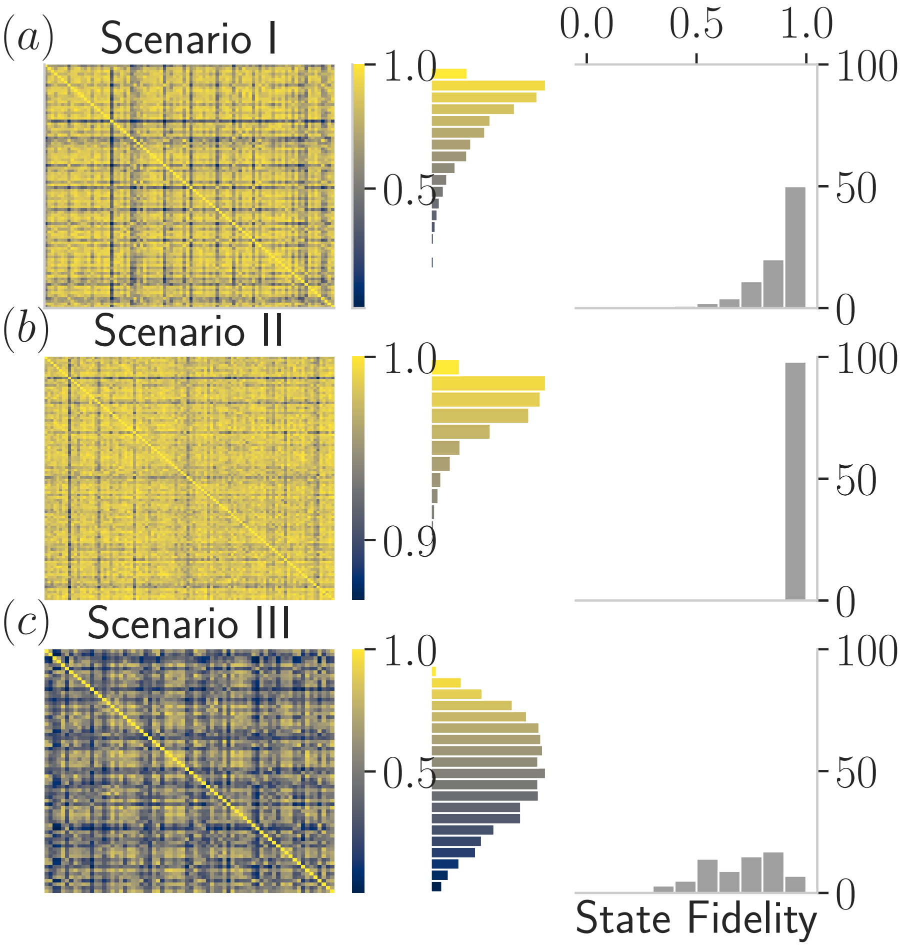

SUPPLEMENTARY FIGURE 4. A visual analysis of the state fidelity behavior in each scenario proposed. The heatmap represents the state fidelity between of each experiment with Dicke state and CMA-ES, then each point reflects the similarity between the quantum states produced by two sets of parameters obtained at the end of the corresponding experiments. The main diagonal is always equal to 1, because it is the fidelity between the quantum states defined by the same set of parameters. The colored histogram in the Y-axis shows how the points in the heatmap are distributed in the state fidelity scale. The gray histogram represents the state fidelity distribution obtained when we compute the fidelity between the quantum states produced by the optimal set of parameters for each experiments and the reference state for each case: (a) Scenario I results. (b) Scenario II results. (c) Scenario III results.

high fidelity with the reference state and are similar between them.

[1] Ali Javadi-Abhari, Matthew Treinish, Kevin Krsulich, Christopher J Wood, Jake Lishman, Julien Gacon, Simon Martiel, Paul D Nation, Lev S Bishop, Andrew W Cross, *et al.*, "Quantum computing with qiskit," arXiv preprint arXiv:2405.08810 (2024).

[2] Shengbin Wang, Peng Wang, Guihui Li, Shubin Zhao, Dongyi Zhao, Jing Wang, Yuan Fang, Menghan Dou, Yongjian Gu, Yu-Chun Wu, *et al.*, "Variational quantum eigensolver with linear depth problem-inspired ansatz for solving portfolio optimization in finance," arXiv preprint arXiv:2403.04296 (2024).

[3] Chandra Sekhar Mukherjee, Subhamoy Maitra, Vineet Gaurav, and Dibyendu Roy, "Preparing dicke states on a quantum computer," IEEE Transactions on Quantum Engineering **1**, 1–17 (2020).

[4] Michael A Nielsen and Isaac L Chuang, *Quantum computation and quantum information* (Cambridge university press, 2010).

[5] Steven Weinberg, *Lectures on quantum mechanics* (Cambridge University Press, 2015).

[6] Samson Wang, Enrico Fontana, Marco Cerezo, Kunal Sharma, Akira Sone, Lukasz Cincio, and Patrick J Coles, "Noise-induced barren plateaus in variational quantum algorithms," Nature communications **12**, 6961 (2021).
[7] Abhinav Kandala, Kristan Temme, Antonio D Corcoles, Antonio Mezzacapo, Jerry M Chow, and Jay M Gambetta, "Extending the computational reach of a noisy superconducting quantum processor," arXiv preprint arXiv:1805.04492 (2018).
[8] Tudor Giurgica-Tiron, Yousef Hindy, Ryan LaRose, Andrea Mari, and William J Zeng, "Digital zero noise extrapolation for quantum error mitigation," in *2020 IEEE International Conference on Quantum Computing and Engineering (QCE)* (IEEE, 2020) pp. 306–316.
[9] Youngseok Kim, Christopher J Wood, Theodore J Yoder, Seth T Merkel, Jay M Gambetta, Kristan Temme, and Abhinav Kandala, "Scalable error mitigation for noisy quantum circuits produces competitive expectation values," Nature Physics **19**, 752–759 (2023).
[10] Ewout Van Den Berg, Zlatko K Minev, Abhinav Kandala, and Kristan Temme, "Probabilistic error cancellation with sparse pauli–lindblad models on noisy quantum processors," Nature physics **19**, 1116–1121 (2023).
[11] Riddhi S Gupta, Ewout Van Den Berg, Maika Takita, Diego Riste, Kristan Temme, and Abhinav Kandala, "Probabilistic error cancellation for dynamic quantum circuits," Physical Review A **109**, 062617 (2024).
[12] Sergei Filippov, Matea Leahy, Matteo AC Rossi, and Guillermo García-Pérez, "Scalable tensor-network error mitigation for near-term quantum computing," arXiv preprint arXiv:2307.11740 (2023).
[13] Youngseok Kim, Andrew Eddins, Sajant Anand, Ken Xuan Wei, Ewout Van Den Berg, Sami Rosenblatt, Hasan Nayfeh, Yantao Wu, Michael Zaletel, Kristan Temme, *et al.*, "Evidence for the utility of quantum computing before fault tolerance," Nature **618**, 500–505 (2023).
[14] Paul D Nation, Hwajung Kang, Neereja Sundaresan, and Jay M Gambetta, "Scalable mitigation of measurement errors on quantum computers," PRX Quantum **2**, 040326 (2021).
[15] Ewout Van Den Berg, Zlatko K Minev, and Kristan Temme, "Model-free readout-error mitigation for quantum expectation values," Physical Review A **105**, 032620 (2022).
[16] Panagiotis Kl Barkoutsos, Giacomo Nannicini, Anton Robert, Ivano Tavernelli, and Stefan Woerner, "Improving variational quantum optimization using cvar," Quantum **4**, 256 (2020).
[17] Samantha V Barron, Daniel J Egger, Elijah Pelofske, Andreas Bärtschi, Stephan Eidenbenz, Matthis Lehmkuehler, and Stefan Woerner, "Provable bounds for noise-free expectation values computed from noisy samples," Nature Computational Science , 1–11 (2024).
[18] Kuan-Cheng Chen, Tai-Yue Li, Yun-Yuan Wang, Simon See, Chun-Chieh Wang, Robert Wille, Nan-Yow Chen, An-Cheng Yang, and Chun-Yu Lin, "Validating large-scale quantum machine learning: Efficient simulation of quantum support vector machines using tensor networks," Machine Learning: Science and Technology (2024).
[19] Marco Fanizza, Matteo Rosati, Michalis Skotiniotis, John Calsamiglia, and Vittorio Giovannetti, "Beyond the swap test: optimal estimation of quantum state overlap," Physical review letters **124**, 060503 (2020).
